# Right iliac arteriovenous fistula due to aneurysm rupture and left iliac artery aneurysm: hybrid treatment

**DOI:** 10.1590/1677-5449.202200172

**Published:** 2023-10-09

**Authors:** Adalberto Pereira de Araujo, Cristiane Ferreira de Araujo Gomes, Douglas Poschinger-Figueiredo, Carlos Felipe da Silva Delgado, Monica Rochedo Mayall, Flavia Figueira Baltharejo Campanario, Felipe Borges Fagundes

**Affiliations:** 1 Centro Angiocardiológico, Rio de Janeiro, RJ, Brasil.; 2 Universidade Federal do Rio de Janeiro - UFRJ, Rio de Janeiro, RJ, Brasil.; 3 Universidade do Estado do Rio de Janeiro - UERJ, Hospital Universitário Pedro Ernesto - HUPE, Rio de Janeiro, RJ, Brasil.; 4 Hospital Estadual Eduardo Rabello, Rio de Janeiro, RJ, Brasil.; 5 Complexo Hospitalar de Niterói - CHN, Niterói, RJ, Brasil.; 6 Clinic Care - Grupo Geriatics, Niterói, RJ, Brasil.

**Keywords:** arteriovenous fistula, iliac aneurysm, iliac vein, iliac artery, heart failure, aneurysm, ruptured

## Abstract

An arteriovenous fistula (AVF) is an uncommon sequela of spontaneous arterial aneurysm rupture into the adjacent venous system. We describe the case of a 74-year-old patient who underwent endovascular treatment of a right iliac AVF caused by a ruptured common iliac artery (CIA) aneurysm and a distal left CIA aneurysm. Surgery preserved the lumbar and inferior mesenteric arteries because of the need to simultaneously exclude the hypogastric arteries. Dynamic fluid balance phenomena provoked by closure of the AVF are described. The patient had a benign postoperative course with normalization of the severe hemodynamic changes presented prior to the intervention and resolution of respiratory symptoms attributed to pulmonary arterial hypertension.

## INTRODUCTION

Arteriovenous fistula (AVF) is an uncommon sequela of spontaneous rupture of an arterial aneurysm into the adjacent venous system, although spontaneous rupture of an abdominal aortic aneurysm (AAA) or iliac artery aneurysm into adjacent veins is the most common cause of AVF in the abdominal cavity, accounting for 80% of reported cases. Around 3 to 4% of ruptured abdominal aneurysms result in this type of complication.^1^

The clinical findings in aorto-caval AVF or AVF between iliac vessels are similar to those provoked by tricuspid insufficiency, leading to high output heart failure (HF) and pulmonary arterial hypertension (PAH). Local signs include a palpable pulsatile abdominal mass in more than 90% of cases and continuous and diffuse abdominal murmur that increases during systole in 73 to 83% of cases, which are considered pathognomonic.^2-4^

Definitive diagnosis is made with computed tomography angiogram (CTA), magnetic resonance angiography, or angiography.^5^ Treatment can be by laparotomy,, with trans-aneurysmal suture of the AVF, venorrhaphy and lateral arteriorrhaphy, arterial graft with ligature of the vein in cases of fistulas between the iliac vessels, or using endovascular techniques to deploy an endoprosthesis. Mortality ranges from 0 to 55% and is lower in fistulas that involve the iliac vessels.^2,3^

The objective of this report is to describe endovascular treatment of a 74-year-old patient with AVF provoked by rupture of a large right common iliac artery (CIA) aneurysm into the right iliac vein and a distal left CIA aneurysm.

### Part I - clinical situation

A male, 74-year-old ex-smoker with hypertension, chronic obstructive pulmonary disease (COPD), PAH, and congestive HF was admitted to a coronary unit in November 2007 for dyspnea and a tight chest, but was free from acute abdominal pains. He developed acute pulmonary edema and was treated for acute myocardial infarction (AMI). He had a prior history of abdominal aneurysm, treated conservatively due to elevated surgical risk. He had no history of prior trauma. While he was in hospital he was scheduled for a colonoscopy to investigate anemia and eliminated bloody fluid during preparation of the colon. The colonoscopy revealed diverticulosis without signs of bleeding and he was discharged from hospital.

The following month, he was seen in emergency with precordial pain, dyspnea, jugular swelling, anasarca, and a pulsatile tumor in the mesogastrium with lateral expansion, and thrill and 5+/6+ continuous abdominal murmur involving the entire abdomen that increased during systole. Work-up found hematocrit at 31.6%, hemoglobin at 9.5 g/dL, and normal biochemistry and an echocardiogram performed on December 18, 2007, found an ejection fraction of 64% and estimated PAH at 56 mmHg.

**Figure 1:**
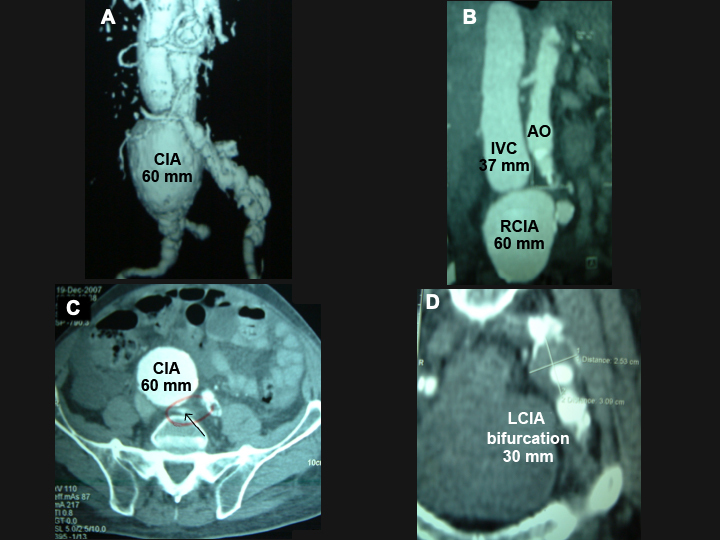
Angiotomography showing (A) and (B) an aneurysm with a diameter of 60 mm along the entire length of the right common iliac artery. Note the significant dilation of the inferior vena cava (37 mm); (C) note the fistula orifice (arrow); (D) aneurysm with a diameter of 30 mm at the bifurcation of the left common iliac artery. AO = aorta; CIA = common iliac artery; IVC = inferior vena cava; LCIA = left common iliac artery; RCIA = right common iliac artery.

The electrocardiogram conducted during typical precordial pains did not reveal acute ischemic changes and there were no myocardial enzyme abnormalities. Arterial and venous Doppler examination of the lower limbs was unremarkable. The CTA showed the abdominal aorta with a diameter of 18mm in all segments; a right CIA aneurysm with a diameter of 60 mm, no proximal or distal neck, ruptured into the ipsilateral iliac vein (AVF aneurysm > right common iliac vein) (Figure 1A and 1C); a 30 mm diameter aneurysm at the bifurcation of the left CIA (Figure 1D); and ascites. His American Society of Anesthesiologists (ASA) surgical risk grade according was IV.

In this case, the treatment options were as follows:

- conservative treatment, considering the patient’s high surgical risk;
- laparotomy for proximal and distal ligature of the common iliac vein and resection of the aneurysms;
- opening the aneurysm to perform trans-aneurysmal suture of the AVF and resection of the aneurysms;
- resection of the aneurysms, repair of the iliac vein and aorto-bi-iliac bypass;
- endovascular treatment with deployment of an endoprosthesis with branches to the hypogastric arteries; or
- embolization of the hypogastric arteries and deployment of an aorto-uni-iliac stent graft with preservation of the lumbar and inferior mesenteric arteries (IMA), followed by a right-to-left crossover femoro-femoral bypass graft.

### Part II - what was done

**Figure 2:**
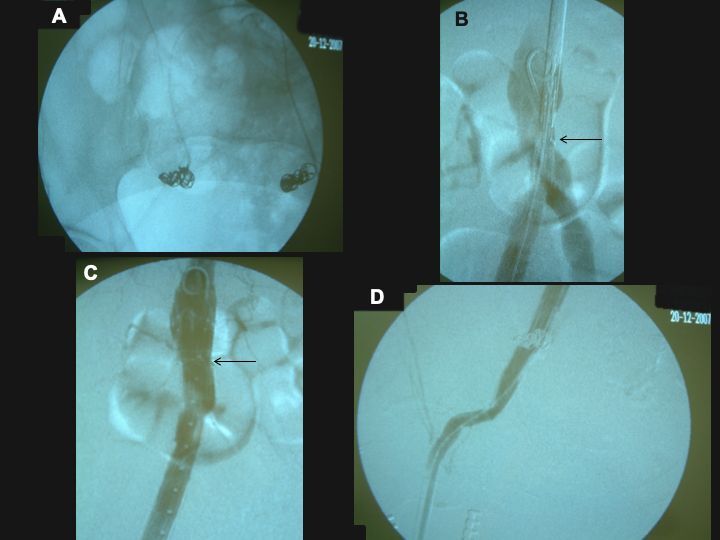
Pelvic radiography and transoperative control angiography. (A) Fibered coils occluding the hypogastric arteries; (B) aorto-uni-iliac stent graft positioned and ready for release, immediately above the bifurcation of the aorta. Observe the marking at the start of the covered portion (arrow); (C) endoprosthesis deployed and encompassing the terminal segment of the aorta below the inferior mesenteric artery, well aligned, and free from leaks. Observe the arrow at the start of the knitted portion; (D) distal extremity of the endoprosthesis at the mid segment of the external iliac artery, with normal patency.

Considering the patient’s advanced age, the elevated surgical risk, and the complexity of open surgery, we opted for endovascular treatment with bilateral inguinal access and approach via the common femoral arteries. Intraoperative angiography confirmed the CTA findings. The procedure comprised embolization of the right hypogastric artery with three 12 x 10 mm fibered platinum coils and embolization of the left hypogastric artery with four 12 x 10 mm fibered platinum coils (Figure 2A), followed by placement, via the right femoral artery, of a 22-12-125 mm aorto-uni-iliac stent graft below the emergence of the inferior mesenteric artery (Figure 2B and 2C, arrows), followed by a 14-37 mm extension to the right external iliac artery (Figure 2D). Next, a 16 x 20 mm exclusion stent was placed into the initial segment of the left external iliac artery, excluding the aneurysm from the left CIA bifurcation. A right-to-left crossover femoro-femoral bypass was then constructed with an 8 mm straight knitted Dacron graft. The procedure was accomplished without complications and lasted 6 hours. Angiography at the end of surgery showed that the fistula had disappeared, the endoprosthesis was well-aligned, and there were no leaks (Figure 2C and 2D). There was no significant bleeding and so no blood transfusions were needed.

The patient’s clinical progress was good during the immediate postoperative period, with 620 mL polyuria during the first 3 hours, on nitroprusside infusion at 15 mL/hour, and no diuretics. Twelve-hour diuresis was 2,390 mL, with infusion of 1,310 mL over the same period.

On the first postoperative day (POD), hematocrit stabilized at 26.4%, with a drop of five points from the preoperative level, with no need for blood transfusion, and creatinine at 0.9 mg/dL. Blood pressure was 140 x 70 mmHg, heart rate was 96 bpm, and the pulse oximetry reading was 96%. Polyuria continued, at 6,120 mL/24h, with infusion of 3,550 mL and no administration of diuretics. Both lower limb edema and anasarca disappeared. The patient was discharged home on the fourth POD with no complaints. During the second postoperative month, his blood tests normalized, with hematocrit at 36%.

**Figure 3:**
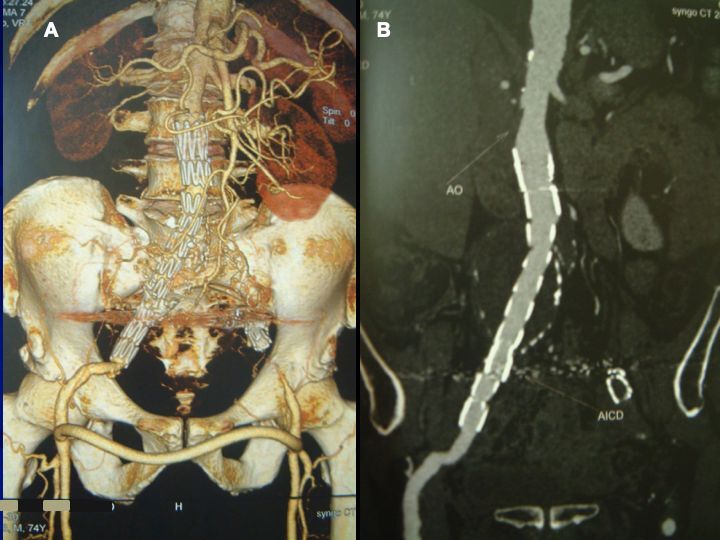
Angiotomography at 6 postoperative months. (A) Aorto-uni-iliac stent graft up to the mid segment of the right external iliac artery, well-aligned. Femoro-femoral cross-over bypass with normal patency and free from kinking or stenosis. Occluding stent in the left external iliac artery; (B) sagittal view confirming patency of the endoprosthesis and the aneurysm with no leakage.

An echocardiogram in the fifth postoperative month showed no more signs of PAH. Urea was at 46 mg/dL and creatinine at 0.9 mg/dL. A CTA during the sixth postoperative month (Figure 3A and 3B) showed the endoprosthesis well-positioned, aligned, and free from leaks, and no flow into the iliac aneurysm, with diameter reduced to 50 mm. The right-to-left crossover femoro-femoral bypass graft was patent, with no signs of stenosis or kinking. During follow-up, at an assessment by the pulmonology team, the respiratory symptoms mimicking COPD had disappeared. The patient remained asymptomatic, with no complaints of gluteal claudication, with all pulses present and strong, and with no edema in the lower limbs. His short, medium, and long-term course was benign. Control CTA showed that the aneurysm had shrunk from its initial 60 mm to 41 mm during the first year. Twelve years after the intervention, the patient complained of dyspnea in response to moderate effort, but his echocardiogram showed pulmonary artery pressure (PAP) of 39 mmHg.

## DISCUSSION

The clinical findings of aortocaval AVF or AVF between iliac vessels are similar to those of tricuspid insufficiency, leading to high output HF. Other signs and symptoms include oliguria, oliguric renal failure, hematuria, and rectal bleeding due to rupture of veins of the rectum and bladder distended by the localized venous hypertension.^6^ Rectal bleeding was seen in the case discussed and precordial pain was also present, simulating AMI.^7^ At literature, we found mention of pulmonary arterial hypertension, which was present in this patient.

Although treatment with open surgery is possible, the endovascular option is now preferred because it is less invasive and it generally involves placement of endoprostheses.^8-10^ Use of this technique was well documented in 2014 by Nakad et al.^11^ who conducted a systematic review of 48 articles, with a total of 54 patients up to 2013. The most common site of fistula was the aortocaval segment. Aortic stents were used in 78% of patients and the technical success rate was 94%. Mortality at 90 days was 10% and half of these deaths were unrelated to the primary pathology.

With the technique adopted, it was necessary to embolize the hypogastric arteries to fit an aorto-uni-iliac stent graft to the external iliac, because when the case was treated there were no endoprostheses with branches to the hypogastric arteries.

Since the aorta was normal, the strategy aimed to avoid exclusion of the lumbar and inferior mesenteric arteries (Figure 2B and 2C), to reduce the risks of pelvic ischemia.

The profuse polyuria seen during the first 24 postoperative hours demonstrated the patient’s intense fluid congestion caused by the presence of the high output AVF, provoking PAH and congestive HF with anasarca.

The pulmonology team had been treating this patient for 2 years as having COPD and PAH, with a PAP of 56 mmHg on the preoperative echocardiogram, and, after additional postoperative investigations, it was concluded that the cause of his respiratory symptoms was PAH. Twelve years and 6 months after the intervention, PAP remained at 39 mmHg. However, the non-availability of control CTAs beyond the sixth month constitutes an objective analytical limitation of the study.

Acquired AFVs involving vessels of the abdominal cavity are a primordial indication for surgery. Treatment with endovascular techniques is undoubtedly the safest option. If exclusion of the hypogastric arteries is unavoidable, it is highly recommendable, whenever possible, to avoid exclusion of the lumbar and inferior mesenteric arteries, as in the present case.^12^ Nowadays, this case would have the ideal indications for use of an endoprosthesis with branches to the hypogastric arteries.

This study was conducted in accordance with the Helsinki Declaration and approved by the Research Ethics Committee at the authors’ institution. This is a new submission, on protocol JVB-2020-0183, after changes requested by peer reviewers had been incorporated. The authors have no conflicts of interest to declare.

## References

[1] Baker WH, Sharzer LA, Ehrenhaft JL (1972). Aortocaval fistula as a complication of abdominal aortic aneurysms. Surgery.

[2] Alexander JJ, Imbembo AL (1989). Aorta-vena cava fistula. Surgery.

[3] Brewster DC, Cambria RP, Moncure AC (1991). Aortocaval and iliac arteriovenous fistulas: recognition and treatment. J Vasc Surg.

[4] Pilan BF, Oliveira AM, Siqueira DED (2014). Treatment of acquired arteriovenous fistula with severe hemodynamic effects: therapeutic challenge. J Vasc Bras.

[5] Schott EE, Fitzgerald SW, McCarthy WJ, Nemcek AA, Sonin AH (1997). Aortocaval fistula: diagnosis with MR angiography. AJR Am J Roentgenol.

[6] Duffy JP, Gardham JRC (1989). Spontaneous aortocaval fistula--preoperative diagnosis and management. Postgrad Med J.

[7] Gordon JB, Newman KD, Marsh JD (1986). Angina pectoris as the initial manifestation of an aortocaval fistula. Am J Med.

[8] Umscheid T, Stelter WJ (2000). Endovascular treatment of an aortic aneurysm ruptured into the inferior vena cava. J Endovasc Ther.

[9] Hetzel G, Gabriel P, Rompel O, Ritter W, Raithel D (2006). Aortocaval fistula after stent-graft repair. J Endovasc Ther.

[10] Cavalcante LP, Bernardes MV, Rocha RD (2013). Tratamento endovascular de fístula aortocaval pós-traumática tardia: relato de caso. J Vasc Bras.

[11] Nakad G, AbiChedid G, Osman R (2014). Endovascular treatment of major abdominal arteriovenous fistulas: a systematic review. Vasc Endovascular Surg.

[12] Iliopoulos JI, Hermreck AS, Thomas JH, Pierce GE (1989). Hemodynamics of the hypogastric arterial circulation. J Vasc Surg.

