# Supplementary material for: Right iliac arteriovenous fistula due to aneurysm rupture and left iliac artery aneurysm: hybrid treatment
Source: J Vasc Bras. 2023 Oct 9;22:e20220017. doi: 10.1590/1677-5449.202200172 (PMC10706151; doi:10.1590/1677-5449.202200172)
Supplement: Translation

# Translation: Fístula arteriovenosa ilíaca à direita por rotura de aneurisma e aneurisma de artéria ilíaca esquerda: tratamento híbrido

**DOI:** 10.1590/1677-5449.202200171

**Authors:** Adalberto Pereira de Araujo, Cristiane Ferreira de Araujo Gomes, Douglas Poschinger-Figueiredo, Carlos Felipe da Silva Delgado, Monica Rochedo Mayall, Flavia Figueira Baltharejo Campanario, Felipe Borges Fagundes

**Affiliations:** 1 Centro Angiocardiológico, Rio de Janeiro, RJ, Brasil.; 2 Universidade Federal do Rio de Janeiro - UFRJ, Rio de Janeiro, RJ, Brasil.; 3 Universidade do Estado do Rio de Janeiro - UERJ, Hospital Universitário Pedro Ernesto - HUPE, Rio de Janeiro, RJ, Brasil.; 4 Hospital Estadual Eduardo Rabello, Rio de Janeiro, RJ, Brasil.; 5 Complexo Hospitalar de Niterói - CHN, Niterói, RJ, Brasil.; 6 Clinic Care - Grupo Geriatics, Niterói, RJ, Brasil.

**Keywords:** fístula arteriovenosa, aneurisma ilíaco, veia ilíaca, artéria ilíaca, insuficiência cardíaca, aneurisma roto

## Abstract

A fístula arteriovenosa (FAV) é uma sequela incomum de rotura espontânea de aneurisma arterial dentro do sistema venoso adjacente. Descrevemos, em um paciente de 74 anos, o tratamento endovascular de FAV ilíaca à direita por aneurisma de artéria ilíaca comum (AIC) roto associado a aneurisma de AIC distal à esquerda, em cuja cirurgia foram preservadas as artérias lombares e mesentérica inferior por necessidade de excluir simultaneamente as artérias hipogástricas. Foram demonstrados os fenômenos na dinâmica do balanço hídrico ocorridos em decorrência da interrupção da FAV. A evolução do paciente foi benigna, com normalização das graves alterações hemodinâmicas que apresentava e com desaparecimento dos sintomas respiratórios atribuídos à hipertensão arterial pulmonar.

## INTRODUÇÃO

A fístula arteriovenosa (FAV) é uma sequela incomum da rotura espontânea de aneurisma arterial dentro do sistema venoso adjacente, mas a rotura espontânea do aneurisma de aorta abdominal (AAA) e do aneurisma de artéria ilíaca nas veias adjacentes é a causa mais comum de FAV da cavidade abdominal, representando 80% dos casos relatados. Dos aneurismas abdominais rotos, cerca de 3 a 4% geram tal complicação^1^ .

Os achados clínicos da FAV aortocava ou entre os vasos ilíacos são similares aos da insuficiência tricúspide, que leva à insuficiência cardíaca (IC) de alto débito e à hipertensão arterial pulmonar (HAP). Os sinais locais são massa abdominal palpável e pulsátil em mais de 90% dos casos e sopro abdominal contínuo e difuso, com acentuação sistólica em 73 a 83% dos casos, considerada patognomônica^2-4^ .

O diagnóstico definitivo é realizado com angiotomografia computadorizada (ATC), angiorressonância ou angiografia^5^ . O tratamento pode ser feito por cirurgia laparotômica, com sutura transaneurismática da FAV, venorrafia e arteriorrafia lateral, enxertia arterial e ligadura da veia em caso de fístula entre os vasos ilíacos, ou por técnica endovascular, com implante de endoprótese. A mortalidade varia de 0 a 55%, sendo menor nas fístulas que acometem os vasos ilíacos^2,3^ .

O objetivo deste relato é descrever o tratamento endovascular em um paciente de 74 anos com FAV provocada por rotura de grande aneurisma de artéria ilíaca comum (AIC) direita na veia ilíaca direita e aneurisma de AIC distal à esquerda.

### Parte I - situação clínica

Um paciente do sexo masculino, com 74 anos, hipertenso, com doença pulmonar obstrutiva crônica (DPOC), HAP, IC congestiva e com passado de tabagismo, foi internado em unidade coronariana em novembro de 2007 por dispneia e opressão precordial, com ausência de dor abdominal aguda. O quadro evoluiu com edema agudo de pulmão e fora, então, tratado como infarto agudo do miocárdio (IAM). Havia história pregressa de aneurisma abdominal, em tratamento conservador por elevado risco cirúrgico. Não havia história de trauma prévio. Durante a internação, foi indicada colonoscopia para investigação de anemia, tendo apresentado eliminação de líquido sanguinolento durante o preparo do cólon. A colonoscopia evidenciou doença diverticular sem sinais de sangramento; assim, seguiu-se a alta hospitalar.

No mês subsequente, foi atendido na emergência com dor precordial, dispneia, turgência jugular, anasarca e tumor pulsátil no mesogástrio com expansibilidade lateral, frêmito e sopro abdominal contínuo de 5+/6+ com acentuação sistólica em todo o abdômen. À análise complementar, observava-se hematócrito de 31,6%, hemoglobina de 9,5 g/dL, bioquímica normal, ecocardiograma realizado em 18/12/2007 com fração de ejeção de 64% e HAP estimada em 56 mmHg.

O eletrocardiograma em vigência de precordialgia típica não apresentou alterações isquêmicas agudas nem anormalidade das enzimas miocárdicas. O Doppler arterial e venoso dos membros inferiores não apresentou alterações. A ATC evidenciou aorta abdominal com 18 mm de diâmetro em todos os segmentos; aneurisma de AIC direita de 60 mm de diâmetro, sem colo proximal e distal, roto na veia ilíaca homolateral (FAV aneurisma > veia ilíaca comum direita) (Figura 1A e 1C); aneurisma de 30 mm de diâmetro na bifurcação da AIC esquerda (Figura 1D); e ascite. O risco cirúrgico de acordo com a American Society of Anesthesiologists (ASA) era IV.

**Figura 1:** Angiotomografia evidenciando (A) e (B) aneurisma de 60 mm de diâmetro em toda a extensão da artéria ilíaca comum direita. Observar a grande ectasia da veia cava inferior (37 mm); (C) observar o pertuito fistular (seta); (D) aneurisma de 30 mm de diâmetro na bifurcação da artéria ilíaca comum esquerda. AIC = artéria ilíaca comum; AO = aorta; VCI = veia cava inferior; AICD = artéria ilíaca comum direita; AICE = artéria ilíaca comum esquerda.

Neste caso, a assunção do caso e a análise das opções terapêuticas seriam:

- tratamento conservador, considerando o alto risco cirúrgico;
- laparotomia para ligadura proximal e distal da veia ilíaca comum e ressecção dos aneurismas;
- abertura do aneurisma para fistulorrafia transaneurismática e ressecção dos aneurismas;
- ressecção dos aneurismas, reparo da veia ilíaca e derivação aorto-bi-ilíaca;
- tratamento endovascular com implante de endoprótese ramificada para as hipogástricas; e
- embolização das artérias hipogástricas e implante de endoprótese aorto-monoíliaca com preservação das artérias lombares e mesentérica inferior, seguida de derivação femorofemoral cruzada direita > esquerda.

### Parte II - o que foi feito

Considerando a idade avançada, o elevado risco cirúrgico e a complexidade da cirurgia aberta, optamos pelo tratamento endovascular com acesso inguinal bilateral e abordagem das artérias femorais comuns. A angiografia intraoperatória confirmou os achados da ATC. O procedimento constou da embolização da artéria hipogástrica direita com três molas de platina fibradas 12 x 10 mm, embolização da artéria hipogástrica esquerda com quatro molas de platina fibradas de 12 x 10 mm (Figura 2A), seguido do implante, via artéria femoral direita, de endoprótese aorto-monoíliaca 22-12-125 mm abaixo da emergência da artéria mesentérica inferior (Figura 2B e 2C, setas), seguido de uma extensão 14-37 mm até a artéria ilíaca externa direita (Figura 2D). A seguir, foi implantado stent oclusor 16 x 20 mm no segmento inicial da artéria ilíaca externa esquerda excluindo o aneurisma da bifurcação da AIC esquerda. Em continuidade, foi feita uma derivação femorofemoral cruzada direita > esquerda com enxerto de Dacron *knitted*, reto, de 8 mm. O procedimento transcorreu sem intercorrências e durou 6 horas. A angiografia ao final da cirurgia mostrou o desaparecimento da fístula, o bom alinhamento da endoprótese e a ausência de vazamento (Figura 2C e 2D). Não houve sangramento relevante e, portanto, hemotransfusão.

**Figura 2:** Radiografia pélvica e angiografia transoperatórias de controle. (A) Molas fibradas ocluindo as artérias hipogásticas; (B) endoprótese aorto-monoilíaca posicionada, pronta para liberação, imediatamente acima da bifurcação da aorta. Observar os marcadores no início da parte recoberta (seta); (C) endoprótese já liberada englobando o segmento terminal da aorta abaixo da artéria mesentérica inferior, bem alinhada, sem vazamento. Observar a seta no início do tecido; (D) extremidade distal da endoprótese no segmento médio da ilíaca externa com perviedade normal.

O paciente evoluiu clinicamente bem no pós-operatório imediato, com poliúria de 620 mL nas primeiras 3 horas, em infusão de nitroprussiato 15 mL/hora, sem uso de diuréticos. A diurese de 12 horas foi de 2.390 mL para uma infusão de 1.310 mL no mesmo período.

No primeiro dia pós-operatório (DPO), o hematócrito estabilizou em 26,4%, com queda de cinco pontos frente ao pré-operatório, sem indicação de hemotransfusão, e creatinina em 0,9 mg/dL. A pressão arterial era de 140 x 70 mmHg, com frequência cardíaca de 96 bpm e oximetria de pulso de 96%. A poliúria persistiu com 6.120 mL/24h para uma infusão de 3.550 mL, sem administração de diurético. Desapareceram o edema dos membros inferiores e a anasarca. A alta para residência foi dada no quarto DPO, sem queixas. No segundo mês pós-operatório, o hemograma normalizou, com hematócrito em 36%.

O ecocardiograma do quinto mês pós-operatório mostrou não haver mais sinais de HAP. A ureia estava em 46 mg/dL, e creatinina, em 0,9 mg/dL. A ATC do sexto mês pós-operatório (Figura 3A e 3B) mostrou a endoprótese bem posicionada, alinhada, sem vazamento, aneurisma de ilíaca sem fluxo, com redução do diâmetro para 50 mm. O enxerto femorofemoral cruzado direita > esquerda encontrava-se pérvio, sem sinais de estenose ou acotovelamento. No acompanhamento e na avaliação da pneumologia, verificou-se o desaparecimento dos sintomas respiratórios que mimetizavam DPOC. O paciente seguiu assintomático, sem queixa de claudicação glútea, com pulsos universais e amplos e sem edema nos membros inferiores. A evolução no curto, médio e longo prazos foi benigna. O aneurisma reduziu dos 60 mm iniciais para 41 mm na ATC de controle no primeiro ano. Após 12 anos da intervenção, houve queixa de dispneia a médios esforços, mas o ecocardiograma evidenciou pressão de artéria pulmonar (PAP) de 39 mmHg.

**Figura 3:** Angiotomografia de 6 meses pós-operatório. (A) Endoprótese aorto-monoilíaca até o segmento médio da artéria ilíaca externa direita, bem alinhada. Derivação femorofemoral cruzada com perviedade normal, sem acotovelamento ou estenose. Stent oclusor em artéria ilíaca externa esquerda; (B) imagem sagital confirmando a perviedade da endoprótese e o aneurisma sem vazamento.

## DISCUSSÃO

Os achados clínicos da FAV aortocava ou entre os vasos ilíacos são similares aos da insuficiência tricúspide que levam à IC de alto débito. Outros sinais e sintomas são a oligúria, a insuficiência renal oligúrica, a hematúria e o sangramento retal por rotura das veias distendidas do reto e da bexiga em consequência da hipertensão venosa regional^6^ . O sangramento retal ocorreu no caso em discussão, assim como a precordialgia, simulando IAM^7^ . Na literatura, encontramos menção de hipertensão arterial pulmonar, a qual esteve presente neste paciente.

Embora o tratamento possa ser feito por cirurgia aberta, a opção endovascular tem sido preferida por ser menos invasiva e geralmente é realizada com implante de endopróteses^8-10^ . O emprego dessa técnica foi bem constatado por Nakad et al.^11^ , que, em 2014, fizeram uma revisão sistemática em 48 artigos, totalizando 54 pacientes até 2013. O local mais comum da fístula foi o segmento aortocaval. Os stents aórticos foram utilizados em 78% dos pacientes, e o sucesso técnico foi de 94%. A mortalidade em 90 dias foi de 10%, e metade das mortes não estavam relacionadas à patologia primária.

Na técnica adotada, foi necessário embolizar as artérias hipogástricas para implantar uma endoprótese aorto-monoilíaca externa, porque naquela época ainda não havia no mercado as endopróteses ramificadas para artérias hipogástricas.

Como a aorta era normal, a estratégia visou evitar a exclusão das artérias lombares e mesentérica inferior (Figura 2B e 2C) para diminuir os riscos de isquemia pélvica.

A profusa poliúria ocorrida nas primeiras 24 horas de pós-operatório demonstrou a intensa congestão hídrica a que o paciente estava submetido pela presença da FAV de alto débito, provocando HAP e IC congestiva com anasarca.

A pneumologia, que já tratava o paciente há 2 anos como portador de DPOC e HAP, com PAP de 56 mmHg em ecocardiograma pré-operatório, após investigações adicionais pós-operatórias, concluiu que a causa dos sintomas respiratórios era HAP. A PAP após 12 anos e 6 meses da intervenção manteve-se em 39 mmHg; entretanto, a indisponibilidade de ATCs de controle além do sexto mês figura como uma limitação analítica objetiva deste estudo.

As FAVs adquiridas dos vasos da cavidade abdominal têm indicação cirúrgica primordial. O tratamento por técnica endovascular é certamente o mais seguro. Caso a exclusão das hipogástricas seja imperiosa, é altamente recomendável, quando possível, evitar a exclusão das artérias lombares e mesentérica inferior, como feito neste caso^12^ . Na atualidade, o caso em referência teria indicação ideal para implante de endoprótese ramificada para as hipogástricas.

O estudo foi conduzido de acordo com a Declaração de Helsinki e aprovado pelo Comitê de Ética em Pesquisa da instituição a que se vinculam os autores. Trata-se de nova submissão, correspondente ao protocolo JVB-2020-0183, realizada após os ajustes solicitados pelos revisores. Não há conflito de interesse a ser declarado pelos autores.
